# ELISA measurement of specific non-antigen-bound antibodies to Aβ1-42 monomer and soluble oligomers in sera from Alzheimer's disease, mild cognitively impaired, and noncognitively impaired subjects

**DOI:** 10.1186/1742-2094-8-93

**Published:** 2011-08-09

**Authors:** Andrea C Klaver, Mary P Coffey, Lynnae M Smith, David A Bennett, John M Finke, Loan Dang, David A Loeffler

**Affiliations:** 1 Department of Neurology Research, William Beaumont Hospital Research Institute, Royal Oak, MI 48073, USA; 2 Department of Biostatistics, William Beaumont Hospital Research Institute, Royal Oak, MI 48073, USA; 3 Rush Alzheimer's Disease Center, Rush University Medical Center, Chicago, IL 60612, USA; 4 Department of Neurological Sciences, Rush University Medical Center, Chicago, IL 60612, USA; 5 Department of Chemistry, Oakland University, 2200 Squirrel Road, Rochester, MI 48309, USA; 6 Eye Research Institute, Oakland University, 2200 Squirrel Road, Rochester, MI 48309, USA

## Abstract

**Background:**

The literature contains conflicting results regarding the status of serum anti-Aβ antibody concentrations in Alzheimer's disease (AD). Reduced levels of these antibodies have been suggested to contribute to the development of this disorder. The conflicting results may be due to polyvalent antibodies, antibody "masking" due to Aβ binding, methodological differences, and/or small sample sizes. The objectives of this pilot study were to compare serum anti-Aβ antibody concentrations between AD, mild cognitive impairment (MCI), and elderly noncognitively impaired (NCI) subjects while addressing these issues, and to perform power analyses to determine appropriate group sizes for future studies employing this approach.

**Methods:**

Serum antibodies to Aβ1-42 monomer and soluble oligomers in AD, MCI, and NCI subjects (10/group) were measured by ELISA, subtracting polyvalent antibody binding and dissociating antibody-antigen complexes. Differences in mean antibody levels were assessed for significance with repeated measures ANOVA using restricted maximum likelihood estimation, using Tukey-Kramer tests and confidence intervals for multiple comparisons. Spearman's rank correlation was used to determine associations between anti-monomer and anti-oligomer antibody concentrations. Estimated sample sizes required to detect effects of various sizes were calculated.

**Results:**

There were no significant differences between groups for mean anti-Aβ antibody levels, although these tended to be higher in AD than NCI specimens. Estimated group sizes of 328 and 150 for anti-Aβ monomer and oligomer antibodies, respectively, would have been required for 80% power for significance at 0.05 for a 25% increase in the AD mean relative to the NCI mean. Serum antibody concentrations to Aβ monomer and oligomers were strongly associated (correlations: 0.798 for undissociated sera, 0.564 for dissociated sera). Antibody-antigen dissociation significantly increased anti-Aβ monomer but not anti-Aβ oligomer antibody levels.

**Conclusions:**

The findings in this pilot study are consistent with relatively similar concentrations of specific, non-antigen-bound antibodies to Aβ1-42 monomer and soluble oligomers in AD, MCI, and NCI sera. The differences between groups for these antibodies would have required approximate group sizes of 328 and 150, respectively, for a high probability for statistical significance. These findings do not support the hypothesis that reduced levels of anti-Aβ antibodies might contribute to AD's pathogenesis.

## Background

Amyloid-beta (Aβ), the major plaque-associated protein in the Alzheimer's disease (AD) brain, has become the main target for AD therapy since the formulation of the "amyloid hypothesis" [1]. The significance of serum antibodies to Aβ in AD is unclear, because these antibodies have been reported to be decreased [2-7], unaltered [8-12], or increased [13-17] in this disorder. These studies are summarized in Table 1. Some investigators have suggested that reduced levels of anti-Aβ antibodies may contribute to the pathogenesis of AD [18,19].

**Table 1:** Summary of previous studies

Study	Specimens	Results
Hyman et al., 2001	Plasma: 82 AD, 271 NCI	No differences between groups (ELISA)

Weksler et al., 2002	Serum: 19 AD, 33 NCI	Decreased AD anti-Aβ levels (ELISA)

Nath et al., 2003	Serum: 16 AD, 31 NCI	Anti-Aβ higher in AD patients

Gruden et al., 2004	Serum: 17 AD, 15 NCI	Increased anti-Aβ25-35 oligomer antibodies in AD patients (ELISA)

Baril et al., 2004	Serum: 36 AD, 34 NCI	No differences between groups (ELISA)

Mruthinti et al., 2004	Plasma: 33 AD, 42 NCI	Anti-Aβ antibodies significantly (4-fold) increased in AD plasma (ELISA)

Moir et al., 2005	Plasma: 59 AD, 59 NCI	No differences for anti-Aβ monomer antibodies; decreased AD levels for anti-Aβ oligomer levels (ELISA)

Brettschneider et al., 2005	Serum: 96 AD, 30 NCI	Anti-Aβ levels decreased in AD (immunoprecipitation assay)

Jianping et al., 2006	Serum: 20 AD, 20 NCI	Decreased AD anti-Aβ levels (ELISA) and avidity

Song et al., 2007	Serum: 153 AD, 193 NCI	Decreased AD anti-Aβ levels (ELISA)

Gruden et al., 2007	Serum: 48 AD, 28 NCI	Increased anti-Aβ25-35 oligomer antibodies in AD patients (ELISA, dot blot)

Gustaw et al., 2008	Serum: 23 or 35 AD (assays performed in two laboratories), 35 NCI	Anti-Aβ levels consistently increased in AD vs. controls only after dissociation

Xu et al., 2008	Plasma: 113 AD, 205 NCI	No differences between groups (plaque immunoreactivity)

Britschgi et al., 2009	Plasma: 75 AD, 36 NCI	No differences between groups (Aβ microarrays)

Sohn et al., 2009	Serum: 136 AD, 210 NCI	Anti-Aβ decreased in AD patients (ELISA)

Gustaw-Rothenberg et al., 2010	Serum: 25 AD < 1 year, 18 NCI, 27 AD > 1 year	Anti-Aβ increased in both AD groups (ELISA) vs. NCI, before and after dissociation

Summary of previous studies in which serum anti-Aβ antibodies have been measured. (AD = Alzheimer's disease; NCI = aged noncognitively impaired)

In previous studies [20,21] we used enzyme-linked immunosorbent assay (ELISA) to measure antibodies to Aβ1-42 monomer and soluble oligomers in intravenous immunoglobulin (IvIg) preparations. IvIg preparations consist of pooled and purified plasma immunoglobulins (> 95% IgG) from thousands of clinically normal individuals. These drugs are being evaluated as a possible treatment for AD; encouraging results were obtained in two clinical trials in which IvIg was administered to AD patients [22,23] and a multi-site phase 3 trial is in progress. In our ELISA studies we found that in addition to IvIg's binding to Aβ-coated wells, it also bound extensively to wells coated with buffer or with an irrelevant protein, bovine serum albumin (BSA). We referred to this as nonspecific binding [20,21] and concluded that it should be subtracted from IvIg's binding to Aβ-coated wells to accurately calculate specific anti-Aβ antibody concentrations. A subsequent study [24] found this binding to be mediated by IgG's Fab fragments and therefore referred to it as "polyvalent." Among previous studies comparing serum anti-Aβ levels between AD patients and aged normal controls, in only one study [3] was this binding subtracted from total antibody binding to Aβ. The conflicting results for anti-Aβ serum antibodies in AD may be due in part to failure to account for this binding. Other reasons could include binding of anti-Aβ antibodies by serum Aβ (antibody "masking"), which could reduce ELISA detection of these antibodies [25], incorrect diagnosis of some study subjects (clinical diagnosis of AD is about 88-90% accurate [26,27]), differences in preparation of the Aβ conformations used to detect antibody binding and/or other methodological differences, and the small sample sizes used in some studies. In previous ELISA studies comparing these antibodies in AD subjects vs. normal controls, only Moir et al. [3], Gruden et al. [14,15], and Nath et al. [13] measured antibodies to Aβ soluble oligomers, which are thought to initiate AD-type pathology [28], and only Gustaw et al. [16] and Gustaw-Rothenberg et al. [17] performed antibody-antigen complex dissociation. None of the studies performed both subtraction of polyvalent binding and dissociation of antibody-antigen complexes, nor did any of the studies confirm clinical diagnoses with post-mortem examinations or perform power analyses.

The objectives of this pilot study were therefore to compare serum antibody levels to Aβ1-42 soluble conformations between AD patients, subjects with mild cognitive impairment (MCI), and aged noncognitively impaired (NCI) individuals, incorporating all of these procedures, and to perform power analyses on the resulting data to obtain estimates of appropriate group sizes for future studies using this approach. Our findings suggest that relatively similar levels of specific, non-antigen-bound antibodies to soluble Aβ1-42 conformations are present in AD, MCI, and NCI sera. Large numbers of samples (estimated group sizes: 328 and 150 for anti-Aβ monomer and oligomer antibodies, respectively) would be required for a high probability of achieving statistical significance for the between-group differences with this approach.

## Methods

### Serum samples

Serum samples were obtained from the Rush Alzheimer's Disease Center (Chicago, IL) from individuals whose diagnosis on the basis of post-mortem clinical review was AD, MCI, or NCI. MCI subjects had only one impaired cognitive domain and no other apparent cause of cognitive impairment. AD patients had no other apparent cause of cognitive impairment. These individuals were participants in the Rush Memory and Aging Project, a community-based, longitudinal clinical-pathologic study of aging and AD. Details of this project were published previously [29]. The study was approved by the Institutional Review Board of Rush University Medical Center and was given exempt status by Beaumont's Human Investigation Committee. Subject summary statistics are shown in Table 2.

**Table 2:** Subject summary statistics by group (based upon post-mortem clinical review).

Diagnosis	Gender	Age at Death (yrs)	PMI (hrs:mins)	ApoE Alleles	Anti-Inflammatory Usage
NCI	2 male 8 female	89.46 ± 1.32	6:21 (3:40, 62:24)	E2E3: 2 E3E3: 6 E3E4: 1*	6 yes, 4 no

MCI	3 male 7 female	89.73 ± 1.41	4:43 (2:55, 20:30)	E2E2: 1 E2E3: 3 E3E3: 3 E3E4: 3	6 yes, 4 no

AD	8 male 2 female	89.55 ± 1.39	4:22 (1:30, 13:35)	E2E3: 1 E3E3: 5 E3E4: 4	8 yes, 2 no

Subject ages are reported as means ± SEM, while PMI values are shown as medians with minimum and maximum values in parentheses. Gender distribution was significantly different between groups (chi square p = 0.020) with the AD group having more males than the other groups. There were no statistically significant differences between groups for age, PMI, frequency of expression of the different apoE alleles, or use of anti- inflammatory medications. ApoE status was unknown for one NCI subject. (AD = Alzheimer's disease; NCI = aged noncognitively impaired; MCI = mild cognitive impairment; ApoE = apolipoprotein E; PMI = post-mortem interval)

### Aβ1-42 monomer and soluble oligomer preparations

Aβ monomer was prepared as described previously [20,21,30]. Aβ1-42 (0.5 mg; AnaSpec, San Jose, CA) was disaggregated by resuspending in 0.25 ml trifluoroacetic acid (TFA, Sigma-Aldrich, Inc., St. Louis, MO) followed by hexafluoro-2-propanol (HFIP, Sigma-Aldrich). It was aliquoted into eppitubes (20 μl/tube), dried overnight (16-20 hr) at room temperature in a fume hood, and stored at -20°C. The Aβ was resuspended in HPLC-grade water adjusted to pH 3.0 with TFA (1 μl TFA per 10 ml HPLC H_2_O). 0.6 ml TFA water was added to an Aβ-containing eppitube, and after thorough vortexing, this was put on ice in a separate tube. The procedure was repeated twice more on the same eppitube, yielding 1.8 ml of Aβ in TFA water. Tris base (21.8 mg) was added to bring the Tris concentration to 100 mM, and 3.8 μl of 12.1 N HCl was added to adjust the pH to 8.8. The preparation was centrifuged (11,752 × g, 5 min), passed through a 0.2 μm filter, and used immediately. The protein concentration of the filtered preparation was 6 μg/ml with the Bio-Rad Protein Assay (Bio-Rad Laboratories, Hercules, CA).

Aβ oligomers were also produced as described previously [20,30]. 4.8 μl of 1% NH_4_OH (AnaSpec) was added to an eppitube of disaggregated Aβ, and after brief vortexing, the tube sat for one min. The contents of the tube were then transferred sequentially to two more Aβ eppitubes, following this same procedure each time. The preparation was water bath sonicated for 4 min, then incubated for one hr at room temperature. After dilution in phosphate buffered saline (PBS; 0.01 M, pH 7.4, with 0.02% azide) to a final concentration of 58 μg/ml, it was used immediately or stored at 4°C for up to one week.

### Western blots of Aβ conformations

Western blots of Aβ monomer and soluble oligomer preparations were performed under both reducing/denaturing and native conditions as described previously [20,30] using 4-20% Tris-HCl Ready Gels (Bio-Rad Laboratories, Hercules, CA). The molecular weight standards for the native gels were from Sigma-Aldrich's Non-Denaturing Molecular Weight Kit (cat. # MWND500). After electrophoresis, the proteins were transferred to Westran S PVDF membranes (Whatman International Ltd., Maidstone, UK). The membranes were then blocked with 10% non-fat dry milk in 0.01 M PBS, pH 7.4 for one hr at room temperature. Membranes were incubated overnight at 4°C with agitation in mouse monoclonal anti-Aβ(1-16) 6E10 (Covance Research Laboratories, Berkeley, CA; 1:5,000 dilution). After incubation in horseradish peroxidase (HRP)- conjugated anti-mouse IgG (Vector Laboratories, Inc., Burlingame, CA; 1:10,000 dilution) for 1 hr at room temperature, membranes were developed in SuperSignal West Pico chemiluminescent substrate (Thermo Scientific, Rockford, IL). Bands were detected on CL-XPosure film (Thermo Scientific).

### Transmission electron microscopy (TEM)

TEM was performed as previously described [31]. Each sample was spread on a Formvar coated grid (Electron Microscopy Sciences, Fort Washington, PA) and incubated for two hr at room temperature, then rinsed with double distilled water. Samples were then fixed with 1% glutaraldehyde in 100 mM phosphate buffer, pH 7.4 for 10 min, rinsed again with water, and stained with 1% uranyl acetate for 10 min followed by alkaline lead citrate for five min. Images were taken with a Morgagni 268 transmission electron microscope (FEI Company, Hillsboro, OR) equipped with a Hamamatsu digital camera.

### ELISA measurement of serum antibodies to Aβ1-42 monomer and soluble oligomers

**Figure 1:**
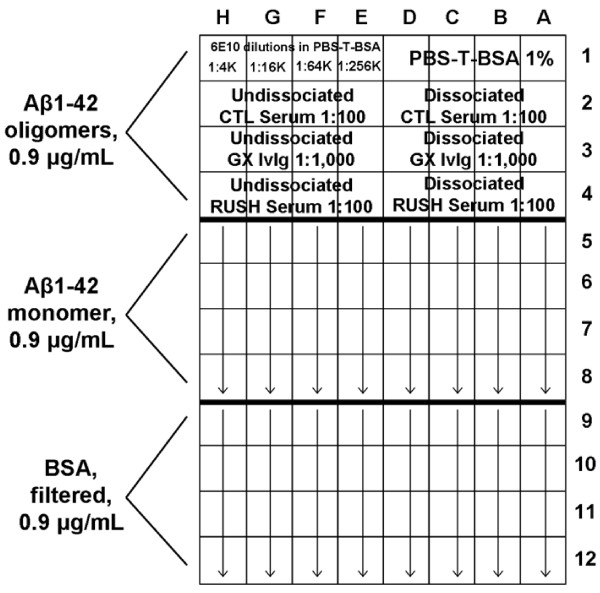
**ELISA plate configuration used to measure specific antibodies to Aβ1-42 monomer and soluble oligomers**. Antibodies to Aβ1-42 (both monomer and soluble oligomers) were measured on a separate ELISA plate for each serum sample. The plate layout for each sample is shown. The mean antibody concentration measured when each serum sample was incubated on BSA-coated wells, representing polyvalent antibody binding, was subtracted from the antibody concentrations measured on wells coated with the soluble Aβ conformations. After calculating the mean anti-monomer antibody concentration of each sample, 30% of this was subtracted from its antibodies to the oligomer preparation to determine its anti- oligomer antibody concentration. An IvIg sample (Gamunex) was included on all plates as a positive control. (CTL serum = normal control serum sample included on all plates to allow normalization of data between plates; Rush serum = experimental serum sample whose anti-Aβ antibody concentrations were being measured; GX = Gamunex Immune Globulin Intravenous (Human), 10%, Talecris Biotherapeutics, Inc., Research Triangle Park, NC).

Antibody concentrations to the Aβ1-42 monomer and soluble oligomer preparations were measured by ELISA in AD, MCI, and NCI serum samples. A separate ELISA plate was required for each serum sample. The plate arrangement is shown in Figure 1. Samples were randomized as to the order in which they were evaluated. A volume of 100 μl was placed in each well for each step of the procedure. The Aβ monomer and soluble oligomer preparations were incubated at 0.9 μg/ml in Tris buffer (0.1 M, pH 8.8) overnight at 4°C on a 96-well Nunc Maxisorp plate (Nalge Nunc International, Rochester, NY). As a "specificity control" the same concentration of bovine serum albumin (BSA, Sigma-Aldrich) in Tris buffer was filtered and placed in adjacent wells. After incubation overnight at 4°C, wells were washed three times with PBS with 0.1% Tween-20 (Sigma-Aldrich) (hereafter, PBS-T; this wash step was repeated after all subsequent incubations). The plate was then treated with SuperBlock (SuperBlock Blocking Buffer in PBS, Thermo Scientific) as per the manufacturer's instructions, followed by addition of antibody-antigen complex dissociated and undissociated serum samples. These samples were diluted 1:100 in PBS (pH 7.2) with 0.1% Tween-20 and 1% BSA (hereafter, PBS-T-BSA) and assayed in quadruplicate. Positive controls were dissociated and undissociated preparations of an IvIg product, Gamunex Immune Globulin Intravenous (Human), 10% (Talecris Biotherapeutics, Inc., Research Triangle Park, NC), diluted 1:1,000. A normal control serum sample from an individual not participating in the Rush Memory and Aging Project was included on all plates to allow data to be normalized between plates. Dissociation of serum antibody-antigen complexes with pH 3.5 dissociation buffer was performed as previously described [20] using the procedure described by Li et al. [25] with slight modifications. To produce the standard curve, four-fold dilutions of mouse monoclonal 6E10 anti-Aβ antibody (1:4,000 [250 ng/ml], 1:16,000 [62.5 ng/ml], 1:64,000 [15.6 ng/ml], and 1:256,000 [3.9 ng/ml]) in PBS-T-BSA were placed in wells previously coated with Aβ monomer, Aβ oligomers, or BSA. Blank wells received PBS-T-BSA at this step. Secondary antisera were biotinylated goat anti-mouse IgG (Vector Laboratories, Inc., Burlingame, CA; 1:1,000 dilution) for the wells previously receiving mouse 6E10 antibody and biotinylated goat anti-human IgG (H + L) (Jackson ImmunoResearch Laboratories, West Grove, PA; 1:1,000 dilution) for wells previously incubated with serum samples. After incubation with streptavidin-alkaline phosphatase (Zymed Laboratories, Invitrogen, Carlsbad, CA; 1:1,000 in PBS-T), para-nitrophenol phosphate (Sigma-Aldrich) was added (5 mg in 40 ml of 1 M diethanolamine buffer, pH 9.8). The plate was read at 405 nm with a Vmax kinetic microplate reader (Molecular Devices Corp., Sunnyvale, CA) until the standard curve OD reached 1.0. Softmax Pro software version 3.0 (Molecular Devices) was used to generate the best-fit plot of the standard curve, using the log-logit option.

### Calculation of serum antibody concentrations to Aβ1-42 monomer and soluble oligomers

To calculate specific anti-Aβ antibody concentrations, the mean antibody concentration measured when each serum sample was incubated on BSA-coated wells was subtracted from the antibody concentrations measured on wells coated with the soluble Aβ conformations. Densitometric analysis of western blots indicated that approximately 30% of the total band intensity in the Aβ oligomer preparation was due to the Aβ monomer band [20]. Therefore, after calculating the mean anti-monomer antibody concentration of each sample, 30% of this was subtracted from its antibodies to the oligomer preparation to determine its anti-oligomer antibody concentration. The antibody levels measured in each experiment were normalized for interassay variation by multiplying them by the overall mean concentration (from all 30 experiments) of anti-Aβ oligomer antibodies in antibody-antigen-dissociated serum from the normal control sample, then dividing by the observed concentration of the anti-Aβ oligomer antibody in this control sample in the experiment. This normalization procedure was based on anti- Aβ oligomer levels in dissociated sera, rather than the other anti-Aβ measurements, because the most consistent findings across experiments were detected for dissociated anti-Aβ oligomer antibody measurements.

### Statistical Methods

Spearman's correlation coefficient was used to assess the association between antibody concentrations to Aβ monomer and oligomeric Aβ using pooled data from all groups and also within each group. Differences in mean antibody levels between groups and between sample preparation methods (either dissociated or undissociated) were assessed with repeated measures ANOVA using restricted maximum likelihood estimation with an appropriate variance structure. Main effects models were used when there was no evidence of interaction. Tukey-Kramer p-values and confidence intervals were used for multiple comparisons as appropriate. The significance of differences between groups was evaluated using one-way ANOVA (for subject age), the Kruskal-Wallis test (for post-mortem intervals [PMI]), and exact versions of Pearson's chi-square tests (for gender, apolipoprotein E [apoE] status, and use of anti-inflammatory medications). P-values ≤ 0.05 were considered statistically significant. All p-values were two-tailed. Statistical analyses were performed using The SAS System for Windows version 9.2.

### Power and sample size analyses

All calculations were based on a significance level of 0.05, with 80% power to detect specified differences using the F test for the group effect from repeated measures ANOVA. The standard deviation of concentration and the mean concentration of anti-Aβ antibodies in NCI sera (averaged between dissociated and undissociated samples) were estimated from the data. The power analysis calculations specified that the mean anti-Aβ antibody concentration in AD subjects would be increased by a given percentage (20%, 25%, 30%, 40%, or 50%) from the antibody concentration in the NCI group. The calculations used NCPASS 2005 software with equal group sample sizes.

## Results

### Western blots of Aβ conformations

Western blots of the Aβ conformations, performed on gels run under both reducing/denaturing and native conditions, were published previously [30]. The Aβ monomer preparation produced a single band in both blots. The blot of the reducing/denaturing gel of the oligomer preparation contained bands corresponding to Aβ monomer, dimer, tetramer, pentamer, and higher-order oligomers. Western blots of the this preparation run on a native gel produced a protein smear in which individual bands were difficult to visualize.

### TEM imaging

**Figure 2:**
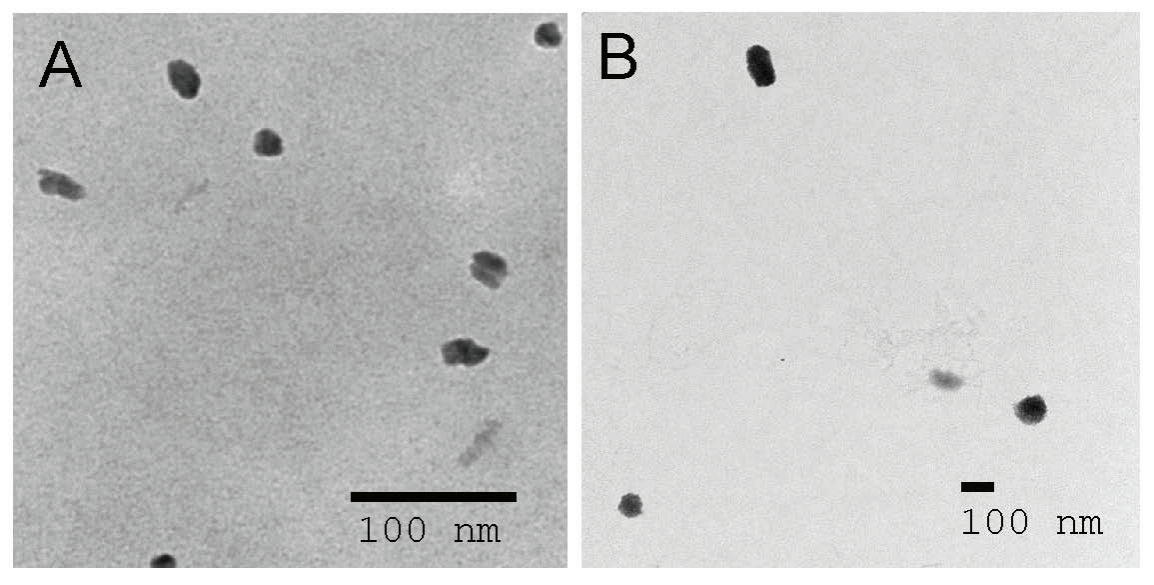
**Transmission electron microscope (TEM) results**. Typical TEM images are shown in Figures 2A and 2B for the Aβ1-42 monomer and oligomer preparations, respectively. The diameters of the spherical structures seen in the Aβ monomer and oligomer preparations were approximately 20 nm and 50-100 nm, respectively.

Spherical structures were present in both the Aβ monomer and Aβ oligomer preparations. The diameter of the spherical structures in the oligomer preparation ranged from 50 to 100 nm while the diameter of the largest spherical structure in the monomer preparation was approximately 20 nm. TEM images are shown in Figure 2.

### Serum anti-Aβ monomer antibodies

**Figure 3:**
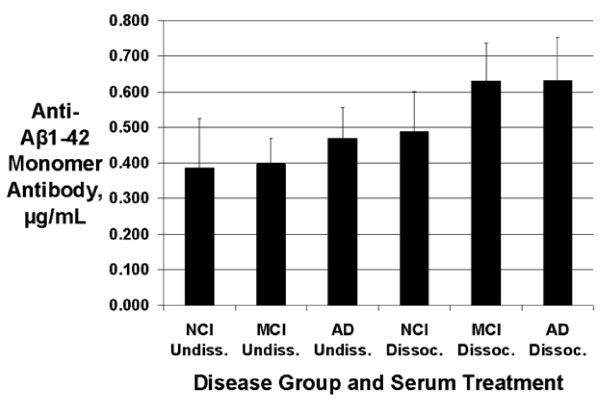
**Serum anti-Aβ1-42 monomer antibody concentrations**. No statistically significant differences were present between group means. For pooled data from all subjects, the antibody levels were significantly increased after antibody- antigen complex dissociation (p = 0.0011), but none of the within-group differences were significant after Tukey-Kramer adjustment of p-values. Data shown are means ± SEM. (AD = Alzheimer's disease; NCI = aged noncognitively impaired; MCI = mild cognitive impairment; Undissoc. = undissociated; Dissoc. = dissociated).

There were no significant differences for serum antibody concentrations to the Aβ monomer preparation between the three groups (p = 0.73 for combined data from undissociated and dissociated serum samples), although the mean concentrations of these antibodies tended to be increased in AD vs. NCI sera (by 20% in undissociated samples and 29% in dissociated samples). 95% Tukey confidence intervals for differences in the mean antibody levels indicated that the possibility of large differences between these groups could not be excluded: MCI - NCI: (-0.280, 0.431); AD - NCI: (-0.243, 0.468); AD - MCI: (-0.318, 0.392). Anti-Aβ monomer antibody levels were significantly increased after antibody-antigen complex dissociation (pooled data from all subjects: p = 0.0011; 95% confidence interval for dissociated - undissociated: [0.073, 0.258]), but none of the within-group differences were statistically significant after Tukey-Kramer adjustment of p-values. Data are shown in Figure 3.

### Serum anti-Aβ oligomer antibodies

**Figure 4:**
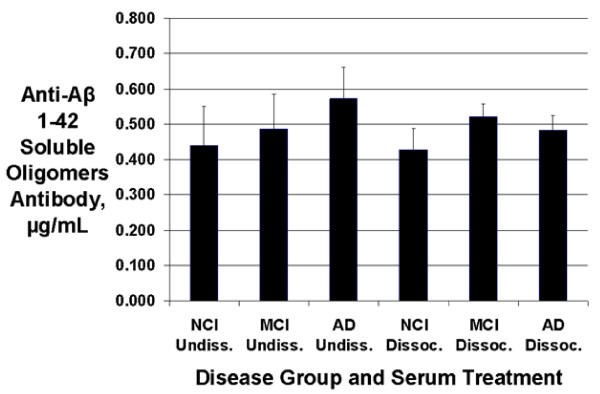
**Serum anti-Aβ1-42 soluble oligomer concentrations**. No statistically significant differences were found between groups or between undissociated and dissociated serum preparations for mean anti-oligomer antibody concentrations. Data shown are means ± SEM. (AD = Alzheimer's disease; NCI = aged noncognitively impaired; MCI = mild cognitive impairment; Undissoc. = undissociated; Dissoc. = dissociated).

Results were generally similar to those for anti-Aβ monomer antibodies. There were no significant differences between the levels of anti-Aβ oligomer antibodies beween AD, MCI, and NCI serum samples (p = 0.58 for pooled data), although the mean levels again tended to be increased in AD vs. NCI sera (30% increase in undissociated sera, 13% increase in dissociated sera), and 95% Tukey confidence intervals for the differences in mean antibody levels indicated that the possibility of large differences between the groups could not be excluded: MCI - NCI: (-0.161, 0.301); AD - NCI: (-0.137, 0.325); and AD - MCI: (-0.207, 0.255). In contrast to the anti-monomer antibodies, antibody- antigen dissociation did not increase mean anti-Aβ oligomer antibody levels (p = 0.65; 95% confidence interval for dissociated - undissociated = (-0.121, 0.072). Data are shown in Figure 4.

### Power analyses

When the population means for serum anti-Aβ monomer antibody concentrations for NCI, MCI, and AD subjects were modeled as 0.440 μg/ml, 0.495 μg/ml, and 0.550 μg/ml, specifying a 25% increase in anti-Aβ monomer antibody levels for AD vs. NCI subjects similar to the findings in the present study, power analysis indicated that 328 samples per group would have been required for 80% probability of statistically significant results at the 0.05 level. For anti-Aβ oligomer antibodies, when the population means for NCI, MCI, and AD were modeled as 0.433 μg/ml, 0.487 μg/ml, and 0.541 μg/ml, resulting in a 25% increase in these antibodies between AD and NCI subjects, 150 samples per group would have been required for 80% probability of significance at the 0.05 level. Tables 3 and 4 indicate the approximate numbers of samples per group that would have been required for 80% probability to achieve significance at the 0.05 level for specified increases in AD vs. NCI antibodies to Aβ monomer and oligomers, respectively, between 20% and 50%.

**Table 3:** Power analysis for anti-Aβ1-42 monomer antibody levels

Specified % Difference Between Means	NCI (μg/mL)	AD (μg/mL)	# Samples Required Per Group (80% power, p < 0.05)
20%	0.440	0.528	512

25%	0.440	0.550	328

30%	0.440	0.572	228

40%	0.440	0.616	129

50%	0.440	0.660	83

The mean concentrations for anti-Aβ monomer antibodies in NCI specimens were determined for pooled data from undissociated and dissociated serum samples. The mean anti-Aβ antibody level in AD subjects was specified to be increased by a given percentage (20-50%) from this NCI antibody concentration, and for each percentage the number of samples per group required to achieve 80% statistical power at a significance level of 0.05 was calculated. Approximately 328 samples per group would have been required to detect statistical significance for the observed differences of 25.7% in this study between NCI and AD means. (AD = Alzheimer's disease; NCI = aged noncognitively impaired)

**Table 4:** Power analysis for anti-Aβ1-42 oligomer antibody levels

Specified % Difference Between Means	NCI (μg/mL)	AD (μg/mL)	# Samples Required Per Group (80% power, p < 0.05)
20%	0.433	0.520	233

25%	0.433	0.541	150

30%	0.433	0.563	104

40%	0.433	0.606	59

50%	0.433	0.650	39

Approximately 150 samples per group would have been required to detect statistical significance for the observed differences of 21.8% in this study between NCI and AD means. (AD = Alzheimer's disease; NCI = aged noncognitively impaired)

### Associations between anti-Aβ monomer and oligomer antibody concentrations

Antibody levels to Aβ monomer and soluble Aβ oligomers were strongly associated. For pooled data from all subjects, Spearman rank correlations were 0.798 for undissociated serum preparations and 0.564 for dissociated preparations. When evaluated for each group, these associations remained positive (data not shown).

### Evaluation of significance for differences between groups for subject variables

There were no significant differences between groups for subject age, apoE status, PMI, or use of anti-inflammatory medications. The gender differences between the groups were statistically significant (p = 0.02) because the majority of the AD group was male (8 males and 2 females) while the other two groups were predominantly females (NCI, 2 males and 8 females; MCI, 3 males and 7 females).

## Discussion

This study used ELISA, with subtraction of polyvalent antibody binding and dissociation of antibody-antigen complexes, to compare the concentrations of serum antibodies to soluble Aβ1-42 conformations between AD, MCI, and NCI subjects who were grouped on the basis of post-mortem clinical review. The between-group differences for serum anti-Aβ levels were not statistically significant. Although the mean levels of these antibodies tended to be increased in AD vs. NCI specimens, large group sizes (estimated at 328 for anti-Aβ monomer antibodies and 150 for anti-Aβ oligomer antibodies) would have been required for a high likelihood that differences of this magnitude would be statistically significant. These sample sizes are considered to be approximate values because they are based on variability estimates from small numbers of samples. Previous studies have suggested that anti-Aβ antibodies may play a protective role in AD, by preventing Aβ's neurotoxicity [32,33], inhibiting development of Aβ soluble oligomers [21], increasing phagocytic clearance of fibrillar Aβ [34], preventing Aβ fibril development [35], and degrading preformed Aβ fibrils [34]. Using procedures to measure specific, non-antigen-bound anti-Aβ antibodies, no evidence was found in the present study for altered levels of these antibodies in AD patients. Because the secondary antibody used to detect anti-Aβ antibodies in the serum samples, biotinylated goat anti-human IgG (H + L), was not IgG-specific, the measurements in the present study represent total serum anti-Aβ antibodies rather than IgG. Our results do not support the hypothesis that decreased concentrations of serum anti-Aβ antibodies may contribute to the pathogenesis of AD.

Some studies have suggested that human anti-Aβ antibodies may recognize conformational epitopes on aggregated forms of Aβ, while not recognizing linear epitopes on monomeric Aβ [12,33,36,37]. However, our IvIg study [20] and the study of Moir et al. with AD and control plasma [3] suggested that these antibodies do include those to Aβ monomer as well as to Aβ oligomers. In the present study, specific antibodies were found in AD, MCI, and NCI sera to both Aβ monomer and oligomer preparations. In an earlier study [30] we evaluated our monomer preparation by western blot after electrophoresis on native gels, immediately after preparation and after storage at 4°C for more than two months. Only one band was seen in each blot, suggesting little, if any, oligomer contamination. The TEM images in the present study also showed clear differences between the 10 nm structures seen in the monomer preparation and the 50 - 100 nm structures observed in the oligomer preparation. These findings suggest that the antibodies measured in the present study to the Aβ monomer preparation were directed to monomer rather than to Aβ oligomers. However, because Aβ monomer may exist in equilibrium with low-order Aβ oligomers [38], the possibility is not ruled out that some of the antibody binding to the Aβ monomer preparation could have been to Aβ oligomers whose concentrations were below the level of detection of western blot.

A further difficulty with regard to differentiating between antibodies to Aβ monomer and oligomers is that anti-monomer antibodies could also recognize Aβ oligomers. The strong association between anti-monomer and anti-oligomer antibody levels in the serum samples in this study raised the issue of whether the two antibody measures may essentially be the same. Depleting the samples of anti-monomer antibodies would not necessarily resolve this issue because this might also remove some anti-oligomer reactivity, if some of the anti-Aβ antibodies bind to both monomers and oligomers. If, in fact, most of the anti-monomer antibodies also recognize oligomers, then after subtracting the ~30% of antibody reactivity to the oligomer preparation which is likely to be due to binding to monomers, little or no reactivity should remain. However, substantial reactivity was still detected. This suggests that at least some of the reactivity was likely to be oligomer-specific.

Previous studies reported that antibody-antigen complex dissociation may allow detection of increased levels of serum anti-Aβ antibodies [16,17,39]. The Aβ conformation to which antibodies were measured in those studies was not stated. In the present study, dissociation increased the measured concentrations of antibodies to Aβ monomer but not to Aβ oligomers. The dissociation procedure used pH 3.5 dissociation buffer to separate antibody-antigen complexes, followed by passage through a 30 kDa molecular weight cutoff filter to remove unbound Aβ. Unlike antibody-antigen dissociation with lower pH (2.5), dissociation at pH 3.5 should not produce artifactual increases in anti-Aβ antibodies or inactivate authentic antibody binding [25]. This procedure should allow removal of Aβ monomer (molecular weight 4.5 kDa) and Aβ oligomers no larger than hexamers (27 kDa), while larger oligomers should be retained. A possible explanation for the lack of an increase in detectable anti-Aβ oligomer antibodies after dissociation is that complexes between anti-Aβ antibodies and larger Aβ aggregates may have re-formed after dissociation, although whether Aβ oligomers are present in serum is unclear. Detection of plasma Aβ oligomers by ELISA was reported by Xia et al. [40], but heterophilic antibodies may have resulted in a false positive signal in that study by crosslinking capture and reporter antibodies, as noted by Sehlin et al. [41]. We found similar false positive results (revealed as such when samples were diluted 1:1 with ELISA Diluent from Mabtech, Inc. [Mariemont, OH], stated by the manufacturer to prevent heterophilic antibody-related false positives) when we attempted to measure total Aβ1-42 in plasma samples from the subjects in this study (data not shown).

Surprisingly, the actual concentrations of specific anti-Aβ antibodies in serum and plasma are unclear. These antibodies have been reported as OD units [5,13,16,24], titers [2,6,9,10,15], and as relative or arbitrary units [3,4,14]. An exception is the study by Storace et al. [39] which reported anti-Aβ antibody levels from dissociated plasma samples from MCI patients and normal controls as both concentrations and OD values. The levels reported in that study ranged from 8.0 to 9.5 μg/ml, higher than the range of 0.4 - 0.6 μg/ml in the present study. The reasons for these differences are unclear. One possibility for this discrepancy is that the concentrations for anti-Aβ antibody concentrations in our study were calculated on the basis of a standard curve using mouse anti-Aβ antibody, whereas Storace et al. used a purified human IgG reference standard. In addition, Storace et al. did not subtract polyvalent antibody binding.

## Conclusions

We report that when specific antibodies to Aβ1-42 monomer and soluble oligomers were measured by ELISA in serum specimens from subjects with post-mortem clinical review diagnoses of AD, MCI, or NCI, no significant differences in these antibody levels were found between groups even after dissociation of antibody-antigen complexes to allow measurement of "free" (non-antigen-bound) antibodies. Further, power analyses on the data indicated that large group sizes (estimated at 328 and 150 for measurements of anti-Aβ monomer and oligomer antibodies, respectively) would have been necessary to achieve a high probability for the between-group differences in these antibody concentrations to achieve statistical significance. These results do not support the hypothesis that decreased levels of these antibodies may contribute to AD pathogenesis.

## List of abbreviations used

AD: Alzheimer's disease; ApoE: apolipoprotein E; BSA: bovine serum albumin; CTL: control; dissoc: dissociated; ELISA: enzyme-linked immunosorbent assay; IvIg: intravenous immunoglobulin; MCI: mild cognitive impairment; NCI: noncognitively impaired; PBS: phosphate-buffered saline; PMI: post-mortem interval; undissoc: undissociated.

## Competing interests

The authors declare that they have no competing interests.

## Authors' contributions

ACK and LMS performed the experimental procedures, collected the data, and assisted in manuscript preparation. MPC performed the data analyses and assisted with manuscript preparation. DAB provided the serum samples and assisted with manuscript preparation. JMF provided guidance with Aβ monomer and oligomer preparation and assisted with manuscript preparation. LD performed the transmission electron microscope studies. DAL directed the research and wrote the manuscript. All authors read and approved the final manuscript.
